# Ultra-high pressure treatment improve the content of characteristic aromatic components of melon juice from the view of physical changes

**DOI:** 10.3389/fnut.2024.1375130

**Published:** 2024-05-17

**Authors:** Xiao Liu, Feng Liang, Bing Su Wang, Fei Yue Ren, Wei Wang, Chao Zhang

**Affiliations:** ^1^Institute of Agri-Food Processing and Nutrition, Beijing Academy of Agriculture and Forestry Sciences, Beijing Key Laboratory of Agricultural Products of Fruits and Vegetables Preservation and Processing, Key Laboratory of Vegetable Postharvest Processing, Ministry of Agriculture and Rural Affairs, Beijing, China; ^2^National Center of Technology Innovation for Grain Industry (Comprehensive Utilization of Edible By-products), Beijing Technology and Business University (BTBU), Beijing, China; ^3^College of Biological and Chemical Engineering, Zhejiang University of Science and Technology, Hangzhou, Zhejiang, China

**Keywords:** ultra-high pressure, melon juice, characteristic aromatic components, surface tension, particle size, thermodynamic parameters

## Abstract

**Introduction:**

The effectiveness of ultra-high pressure (UHP) technology in retaining the flavor of fresh fruit and vegetable juices has been acknowledged in recent years. Along with previously hypothesized conclusions, the improvement in melon juice flavor may be linked to the reduction of its surface tension through UHP.

**Methods:**

In this paper, the particle size, free-water percentage, and related thermodynamic parameters of melon juice were evaluated in a physical point for a deeper insight.

**Results:**

The results showed that the UHP treatment of P2-2 (200 MPa for 20 min) raised the free water percentage by 7,000 times than the other treatments and both the melting enthalpy, binding constant and Gibbs free energy of P2-2 were minimized. This significantly increased the volatility of characteristic aromatic compounds in melon juice, resulting in a 1.2-5 times increase in the content of aromatic compounds in the gas phase of the P2-2 group compared to fresh melon juice.

## 1 Introduction

Melon (*Cucumis melo* L.) is a kind of fruit popular among people all over the world, it belongs to the Cucurbitaceae family, with yellowish-green skin, delicate and juicy flesh, and sweet taste. Melons are rich in vitamin A (β-carotene) and high levels of vitamin C. A 100 g hami melon contains 36.7 mg of vitamin C and 3,382 IU of vitamin A (1). Equivalent to 61.16 and 67.65% of the daily intake of vitamins C and A for Chinese adults (2, 3). China holds the title of being the world’s largest producer and consumer of melons. According to data released by China’s National Bureau of Statistics, the cultivation area of melons in China reached 0.39 million hectares in 2019, and the total output of melons was about 1.36 million tons. Melons, being a typical non-climacteric fruit, face challenges in flavor retention, storage, and transportation, which limits their marketability. Due to limited processing applications, products made from fresh melons are rarely seen other than those sold as fresh fruits in the market. Therefore, the development of melon-based juices in combination with excellent processing technologies may present a promising opportunity.

In recent years, most of the fruit and vegetable juices are processed by using ultra-high temperature (UHT) sterilization technology (4), but since melon is a heat-sensitive fruit, the great damage to the flavor of melon juice caused by excessive temperature change is inevitable (5). Therefore, ultra-high pressure (UHP) technology is considered more suitable for treating melon juice. UHP is a non-thermal treatment technology that achieves pasteurization by introducing the product into a vessel that is subjected to high hydrostatic pressure (6, 7). UHP provides better inactivation of microorganisms and less damage to vitamins and original flavors than conventional thermal treatments. For example, in UHP treated orange juice (8), mango juice (9), peach juice (10), and lychee juice (11), there were no significant differences in terms of odor, taste, and overall acceptability of the UHP juices as compared to untreated fresh juices.

The current research on the effects of UHP on melons has primarily focused on biological and chemical aspects. This includes studies on breeding, genotype screening, bacteriostatic effects, and storage period of melons. Research on the flavor of melon juice affected by UHP has mainly focused on fatty acid metabolic pathways and single flavor characterization, as well as quality characterization using equipment techniques such as electronic nose, electronic tongue, and GC–MS. Fewer investigations have been carried out on the physical properties related to the influence of UHP on the flavor of melon juice. One relevant study involved selecting different pressures of CO to pressurize freshly cut melons before ultrasonic-assisted immersion freezing to improve the freezing effect. Utilizing the effect of ultrahigh pressure on the distribution of water state can help enhance the cavitation effect of ultrasonic waves, thus improving the freezing effect of melons (12, 13). However, there are gaps regarding the connection of ultrahigh pressure affecting the reorganization of intramolecular and intermolecular bonds in fruit juices and changes in water molecules in flavor release.

Since the impact of UHP on the flavor of melon juice from a physical perspective is not fully understood, the research group engaged in a series of speculations and demonstrations regarding the mechanism of UHP’s effect on the flavor of melon juice. The primary conclusion drawn was that UHP can elevate the concentration of volatile and aromatic components by reducing the surface tension of melon juice (14). In response to this conclusion, this paper delved deeper to explore and validate it. This study postulated the effect of UHP on the molecular dynamics of melon juice by examining particle size, low-field nuclear magnetic resonance (LF-NMR), and other indicators of melon juice. Additionally, thermodynamic indices such as differential scanning calorimetry (DSC) and isothermal titration calorimetry (ITC) of melon juice were measured to confirm the mechanism by which UHP enhances the characteristic aromatic components of melon juice.

## 2 Materials and methods

### 2.1 Experiment design

The melons were purchased from a local supermarket in Beijing in the summer of 2021. The melon (*Cucumis melo* L. var. Xizhoumi No. 25) was ellipsoid, light gray, with a light net, weighing about 1.2–2.5 kg per fruit. The flesh was light orange with a soluble solids concentration of 10%.

#### 2.1.1 Control

The surface of the melon was rinsed twice with ice water. Removed the skin and seeds and cut the pulp into cubes in a hygienic processing plant. The pulp was then pulverized using a Philips juicer (HR1861, Philips Co., Ltd., Beijing, China) for 5 min. After removing the foam from the top, the juice was quickly sealed in a 200 mL aluminum foil pouch and stored in a refrigerator at −4°C for subsequent sample determination.

#### 2.1.2 UHP

Fresh melon juice was processed at room temperature in an ultra-high pressure unit (BDS200-FL, Stansted Fluid Power Ltd., UK). The melon juice was divided into four treatment groups that were subjected to different high pressures: (1) 200 MPa for 20 min; (2) 400 MPa for 20 min; and (3) 600 MPa for 20 min. They were designated P2-2, P4-2, and HP6-2, respectively. The holding time did not include pressurization and pressure release times. The system automatically released pressure within 10–20 s after the hold time was reached.

### 2.2 Analysis of volatile compounds

The method was improved according to the method previously screened by the group (14). Headspace solid phase microextraction (HS-SPME) tandem gas chromatography–mass spectrometry (GC–MS) method was used to detect melon juice volatile compounds. A 6 g sample was transferred into a 20 mL headspace glass vial containing 2.0 g NaCl and 10 μL of octanol (30 μg/mL) as an internal standard. Unlike the previous method, in this experiment we used two incubator temperature settings to simulate the normal and complete volatilization of volatile compounds from melon juice. The incubator temperature was set at 50°C and room temperature. After aspiration, the absorbed compounds were thermally desorbed by a GC–MS system at 250°C for 3 min in separation-free mode. Volatile compounds were separated on a DB-5 flexible capillary column. Helium (99.99%) was used as the carrier gas at a constant flow rate of 1.0 mL/min. The initial temperature in the oven was set at 35°C and held for 5 min, then ramped to 150°C at a rate of 4°C/min and held for 3 min, ramped to 190°C at a rate of 8°C/min and held for 1 min, and then ramped to 250°C at a rate of 30°C/min and held for another 5 min. Signal acquisition was performed in full scan mode at 1562 u/s. The mass detector was in electron collision mode (70 eV). The temperature of the ion source was 230°C, the temperature of the transmission line was 250°C, and the temperature of the quadrupole was 150°C. The mass spectra of the detected volatile compounds were identified by comparing them with those in the Mass Spectral Library (NIST17). The concentration of each volatile compound was calculated from the peak area of the internal standard n-octanol at a known concentration (Equation 1).

(1) $$m_{x}=\frac{C_{i}\times V_{i}\times A_{x}}{m_{s}\times A_{i}}\times 1,000$$

Where *C_i_* was the mass concentration of the internal standard compound in μg/mL; *V_i_* was the amount of internal standard added to the sample, 10 μL; ms was the mass of 6 g of the sample; *A_x_* and *A_i_* were the peak areas of the target compounds and the internal standard compounds, respectively; and *m_x_* was the concentration of the target compounds, expressed as μg/kg fresh weight (FW).

### 2.3 Calculation of gas–liquid partition ratios for characteristic aromatic compounds

We assume that in the ideal situation, measurement data under SPME heating were formulated as complete volatilization of the volatile components of melon juice, as the total concentration of the characteristic aroma-presenting components of melon juice in melon juice *CT*. The data from the SPME measurements at room temperature were formulated as the volatilization of the volatile components of melon juice in their natural state, and as the concentration of the characteristic aroma-presenting components of melon juice in the gas, *Cg*. Then the concentration of the characteristic aromatic component of melon juice in the liquid was *Cl* = *CT* - *Cg*.

The gas–liquid partition ratio of the characteristic aromatic components of melon juice was calculated with reference to Equation (2).

(2) $$Y_{x}=\frac{C_{g}}{C_{l}}$$

Where *C_g_* and *C_l_* were the concentrations of the aromatic components in the gas and liquid, respectively, in μg/kg, and the resulting ratio *Y_x_* was the gas–liquid partition ratio of the characteristic aromatic components.

### 2.4 Determinations of particle size

Particle size distribution was determined by laser diffraction using a Laser Particle Sizing Instrument (Microtrac S3500, Microtrac MRB, United States). The particle size analyzer can provide particle size distributions from 0.1 to 1,000 μm. Melon juice was poured into a wet sample dispersion unit containing deionized water and uniformly dispersed with a stirrer subsequently pumped through the optical box. Measurement starts when the sample reaches about 5% occlusion (15). Three measurements were taken for each sample and then averaged.

There were a variety of particle size averages. The labeling symbol was *d (A, B)*, *A* can be a value from 1 to 4, *B* can be a value from 0 to 3, and *A* was > *B* (16). The *d3,2* was the surface area volume mean diameter. The *d4,3* was the volume-weighted average diameter. The *d1,0* was the number-averaged particle size, i.e., the average of the number of particles of different sizes. The size distribution was calculated using the following formula:

The *d50* was the median diameter, which in the cumulative distribution corresponded to the particle size at 50% of the cumulative value (15). This data was automatically generated by the particle size analyzer.

(3) $$d_{1,0}=\sum n_{i}d_{i}/\sum n_{i}$$

(4) $$d_{3,2}=\sum n_{i}{d_{i}}^{3}/\sum n_{i}{d_{i}}^{2}$$

(5) $$d_{4,3}=\sum n_{i}{d_{i}}^{4}/\sum n_{i}{d_{i}}^{3}$$

where *d_i_* was the particle size and *n_i_* was the total number of particles.

### 2.5 Determinations of low-field nuclear magnetic resonance

LF-NMR was performed on a 23 MHz NMR analyzer PQ001 (Newmag Ltd., China). An LF-NMR analysis system, with a main frequency of 23 MHz, a coil diameter of 60 mm, a magnet temperature of 32°C, and a magnet strength of 0.5 T was used in the experiments (17). The experimental parameters were set as 90° pulse time P1 = 18 μs, 180° pulse time P2 = 36 μs, echo time TE = 0.6, spectral width SW = 100 kHz, Echo count = 18,000, repetitive scanning number NS = 4, and sampling repetition time TW = 6,000 Ms. A 5 mL sample of melon juice was weighed for *T2* collection and recording at room temperature. The samples were placed into glass tubes with a diameter of 18 mm, and then the samples were analyzed and measured in an NMR instrument to obtain the exponential decay spectra. Three signals were acquired at a time to observe the stability of the signal amplitude. Finally, the inversion software MultiExp-Inv Analysis was used to obtain the inversion spectra of *T2* and the area data of *T2* relaxation time and its corresponding moisture state of melon juice samples. Measurements were repeated 3 times for each sample to take the average value.

### 2.6 Determinations of rheological property

Both the apparent dynamic viscosity and shear stress were determined by a rheometer Discovery HR-1 (TA Instruments, United States). Optional 40 mm diameter stainless steel parallel plate measurement system with 1 mm spacing between parallel plates (15), the shear rate was set at 10–600 s^−1^. 1 mL of melon juice was placed between the plates and the changes between apparent dynamic viscosity, shear stress and shear rate of melon juice were determined separately. Results were averaged over 3 repeats for each experimental condition.

### 2.7 Determinations of differential scanning calorimetry

Analyses were performed using a Pyris Diamond DSC device (Diamond, Perkin Elmer) injected at a nitrogen flow rate of 20 mL/min. First selected crucibles with no more than 5% difference in liquid crucible weights to spare and recorded the weight of the empty crucibles. Approximately 5 μL of the melon juice sample was placed in a liquid aluminum pot and subsequently placed in the DSC instrument and the following steps were performed (18): (1) Hold for 1.0 min at 20°C, (2) Cool from 20°C to −25°C at 5°C/min, (3) Hold for 2.0 min at −25°C, (4) Heat from −25°C to 20°C at 10°C/min, (5) Hold for 2.0 min at 20°C. Data were selected for analysis in the interval from −10°C to 10°C, complete with the entire melting process of melon juice.

### 2.8 Determinations of isothermal titration calorimetry

Analysis of thermodynamic interactions between ethyl butyrate and melon juice samples using isothermal titration calorimetry (Nano ITC, TA Instruments, United States) (19). Ethyl butyrate and melon juice sample solutions were configured using acetone/citric acid buffer solution. The concentration of ethyl butyrate was 100 mmol/L. Melon juice samples were diluted 10-fold using buffer and filtered through a 0.22 μm filter membrane to a dilution concentration of 10 mmol/L.

Ethyl butyrate was used to titrate the melon juice sample pattern. The initial volume for titration of ethyl butyrate solution was 50 μL (inhalation syringe), and the initial volume for titration of melon juice sample was 350 μL (addition to the sample cuvette). The specific titration conditions were: 20 drops of 2.5 μL each, a titration interval of 200 s, a temperature of 25°C, and a stirring speed of 350 rpm to ensure adequate mixing and equilibration. The blank control was the titration buffer of ethyl butyrate solution.

Binding constants (*Ka*), stoichiometric ratios (*n*), enthalpy changes (*ΔH*), entropy changes (*ΔS*), and Gibbs free energy changes (*ΔG*) were obtained using Multiple Sites model fitting (20).

The Gibbs free energy change (*ΔG*) was calculated using the formula (Equation 6):

(6) ΔG=ΔH−TΔS

### 2.9 Statistical analysis

All the measurements were repeated for 3 times. One-way analysis of variance was conducted on different groups using SPSS Statistics 26.0. The results were shown as mean ± standard deviation at a significance level of *p* < 0.05. The graphs were plotted using by Origin 2021 and Powerpoint.

## 3 Results and discussion

### 3.1 Calculation and analysis of gas–liquid partition ratios of characteristic aromatic components in melon juice

**Figure 1:**
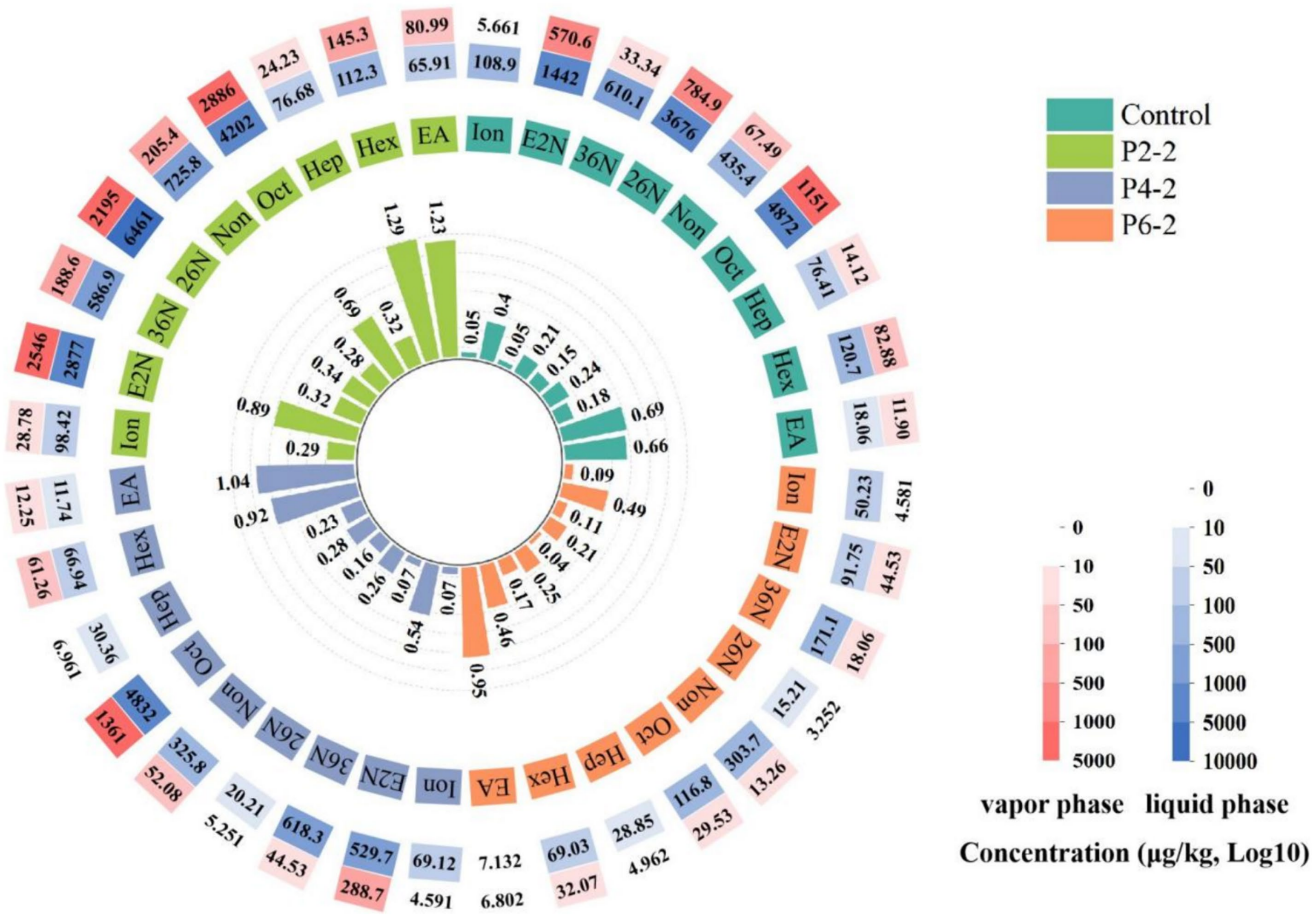
Effect of UHP treatment on the concentration and gas–liquid partition ratio of characteristic aromatic components of melon juice. EA, Ethyl Acetate; Hex, Hexanal; Hep, (E)-2-Heptenal; Oct, Octanal; Non, Nonanal; 26 N, (E,Z)-2,6-Nonadienal; 36 N, (E,Z)-3,6-Nonadienol; E2N, (E)-2-Nonenal; Ion, β-Ionone.

The effect of different pressure levels on the gas–liquid partition ratio of the characteristic aromatic components of melon juice (Figure 1). P2-2 all resulted in a significant increase in the gas–liquid partition ratio of all melon juice characteristic aromatic components. The gas–liquid partition ratios of elevated (E, Z)-3,6-nonadien-1-ol and β-ionone were as much as five times higher than those of the control. It can be seen that the UHP treatment parameter of 200 MPa for 20 min resulted in a significant increase in the volatility of the characteristic aromatic components of melon juice. In addition, the gas–liquid partition ratios of most of the aromatic components decreased significantly with increasing UHP treatment pressure. This may be due to the fact that excessive pressure can lead to structural dissociation of the aromatic component and fission into other components, thus making the concentration of that component lower. In addition, the change in the gas–liquid partition ratio of melon juice was hypothesized to be directly related to its surface tension. In the group’s previous studies, P2-2 significantly reduced the surface tension of melon juice, thereby releasing the characteristic aromatic properties of melon juice (14). This may be due to the fact that the vapor–liquid interfacial properties of purely inhomogeneous systems, such as surface tension, depend on the type of molecule and its molecular structure. And it was usually influenced by the strength of the cohesive forces between molecules, which were determined by the nature of the intermolecular bonds (21).

### 3.2 Effect of ultrahigh-pressure treatment on the particle size of melon juice

**Figure 2:**
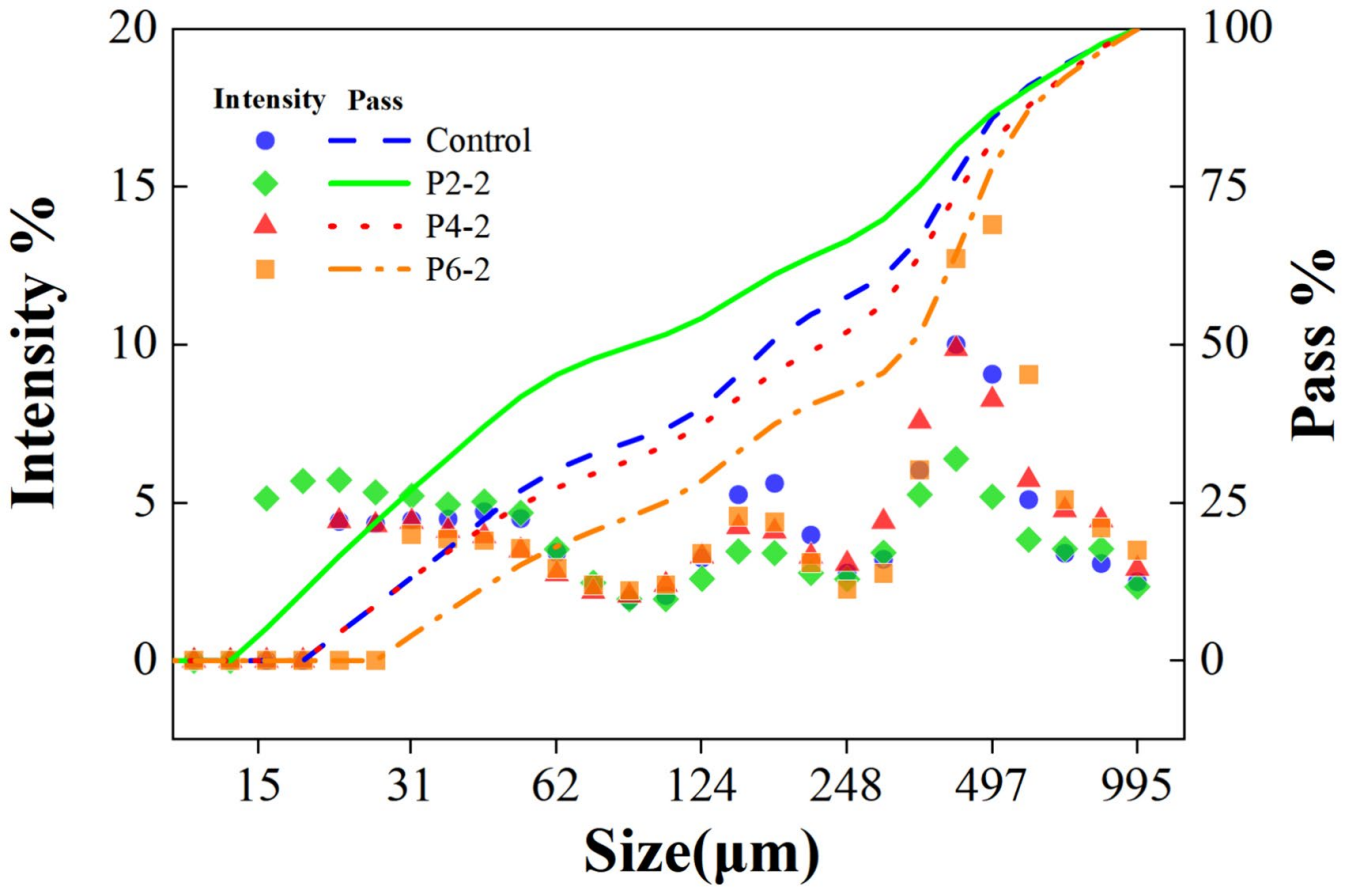
Particle size distribution (PSD) of melon juice under different treatment pressures.

The UHP treatment reduced the average particle size of melon juice (Figure 2). The same results were demonstrated in juices such as highly concentrated tomato suspension (22) and orange juice (23). The presumed reason for this was that the UHP process destroys the cells of the juice and breaks up the fragments into smaller suspended particles (24). Smaller pieces were less susceptible to breakage during processing compared to larger pieces or even whole cells. In addition, in terms of the passage rate of different particle sizes, the P2-2 treatment group had the highest passage rate of particle sizes in the whole region, then it indicated that P2-2 had the best effect on the crushing of melon juice cells, which resulted in the smallest and the most uniform particle sizes (Figure 2). And with the increased pressure, the particle size of melon juice gradually increased and the passage rate was significantly reduced. The presumed reason for this was that the effect of UHP on the destruction of suspended particles appeared to follow an asymptotic behavior, i.e., at higher pressures, the increase in pressure resulted in a smaller change in PSD (25). Similar results were demonstrated in tomato juice (26). In contrast, UHP broke the cells of the juice, which resulted in enhanced volatilization of the characteristic aromatic components of melon juice, thereby increasing the components concentration in the headspace phase. This was consistent with the previous GC–MS results (Figure 1).

Table 1 further showed the detailed changes in *d50*, *d1,0*, *d3,2*, and *d4,3* of melon juice under different high pressure treatments. The intervention of the pressure caused a significant change in the particle size of the melon juice. The *d50* and *d1,0* can reflect the overall particle size and fineness of the juice. P2-2 had the smallest *d50* and *d1,0*, representing its most uniform particles. As the treatment pressure increased, *d50* and *d1,0* also became significantly bigger, indicating that too high a pressure would cause the particles in melon juice to become bigger. The *d3,2* was reported to be affected by smaller particles, while *d4,3* was affected by bigger particles (27). Both small (*d3,2*) and big (*d4,3*) particles of melon juice were increased by UHP treatment. The effect of the increase in P2-2 was the most pronounced, resulting in a maximum of both *d3,2* and *d4,3*. It can be seen that P2-2 caused more intense breaking of melon juice particles. Pressure intensity significantly affected the particle size of melon juice, and different pressure levels had different effects on the particle size of melon juice, with the P2-2 treatment resulting in the most uniform and delicate particles of melon juice (Table 1). And this was similar to the study where nanobubbles significantly reduced the surface tension of the liquid surface (28). With the smaller particle size, the lower the surface tension and the easier the liquid film broke down. And thus the easier the aromatic components of melon juice evaporated.

**Table 1:** Mean particle size of melon juice under different pressure level.

Treatments	*d*_50_	*d*_1,0_	*d*_3,2_	*d*_4,3_
Control	171.20 ± 1.12^c^	276.88 ± 1.04^c^	636.10 ± 2.23^c^	726.99 ± 3.45^c^
P2-2	90.32 ± 0.84^d^	228.75 ± 1.63^d^	670.06 ± 3.25^a^	757.36 ± 1.24^a^
P4-2	222.20 ± 1.62^b^	304.10 ± 2.03^b^	658.34 ± 2.58^b^	742.40 ± 2.23^b^
P6-2	340.30 ± 1.78^a^	369.42 ± 1.03^a^	652.44 ± 2.61^b^	729.82 ± 3.29^c^

Results are presented as mean ± standard deviation (*n* = 3). Different lowercase letters following the values within the same column indicate significant differences (*p* < 0.05).

### 3.3 Effect of ultrahigh-pressure treatment on low-field nuclear magnetism resonance of melon juice

**Figure 3:**
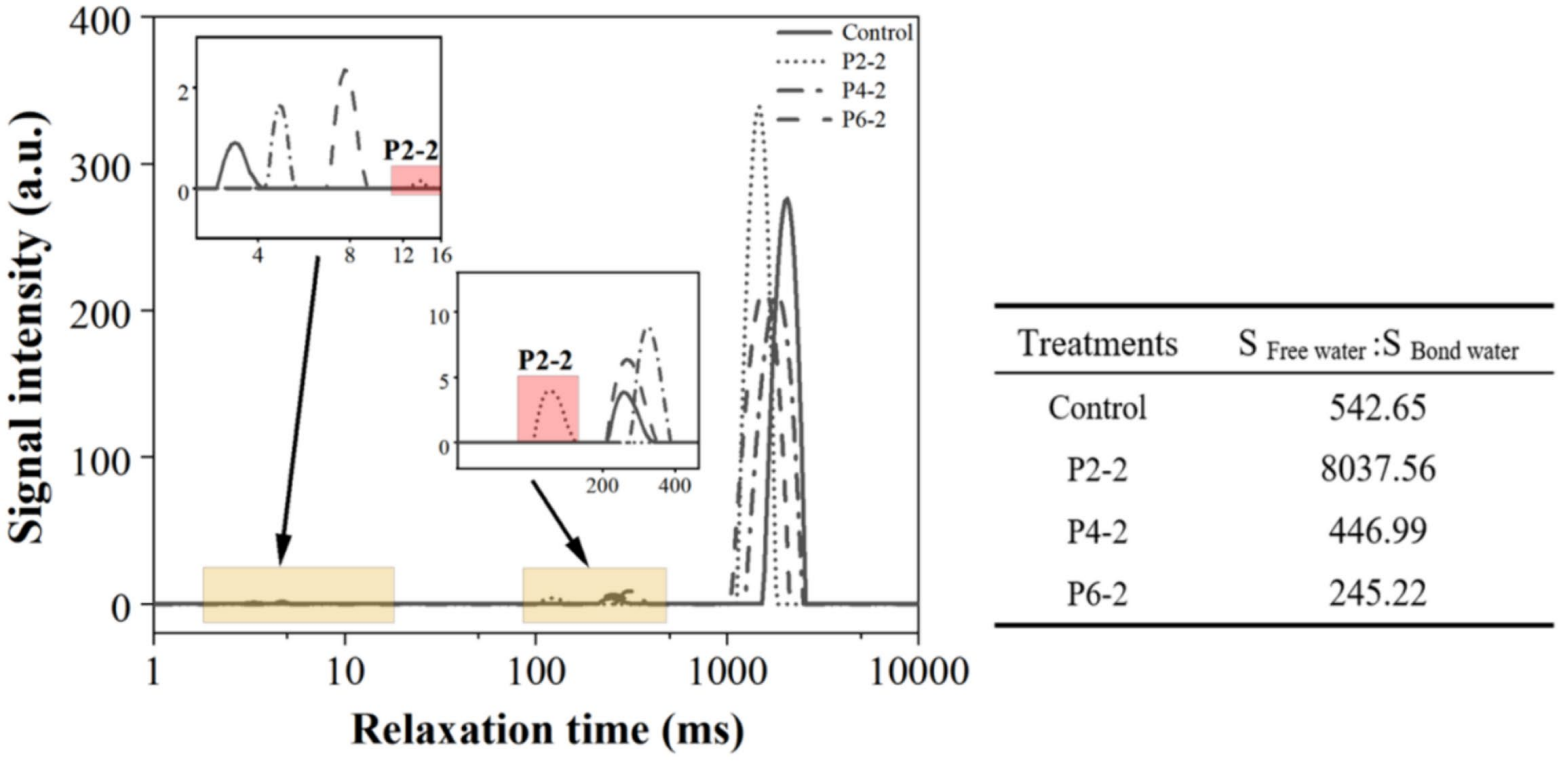
Influence of UHP pressure level on the T_2_ relaxation time of melon juice. The table showed the ratio of free water and bond water area of melon juice. The data in the figure are all calculated as the average value.

In general, *T2* relaxation time can be used as an indicator to monitor water mobility, and water in different environments exhibits different *T2* relaxation properties (17). The transverse relaxation time *T2* measured by LF-NMR reflects the chemical environment of hydrogen protons in the sample. *T2* inversion profiles were obtained by multi-exponential fitting of NMR signal data of melon juice at different high pressures (Figure 3).

As shown in Figure 3, there are three peaks in the plot, from left to right, *T21* (1–15 ms), *T22* (100–400 ms) and *T23* (1,000–3,000 ms). The water molecules of *T21* were defined as “bound water,” the part of water that was most tightly bound to other molecules and cannot rotate in a magnetic field. The water molecules of *T22* were defined as “semi-bound water” and were considered to be bound water which able to rotate in a magnetic field (29). *T23* water molecules were defined as “free water,” which has the molecular mobility of water in aqueous solution and was thought to be present in vesicles, protoplasts, and intercellular spaces in cellular structures (30).

As the drying time increased, both *T2* and the area of each peak changed. The magnitude of *T2* reflected the freedom of water in the sample, and the peak areas reflected the water content of the different moisture. The relaxation time *T2* becomes shorter and the position of the peak on the *T2* spectrum is closer to the left when the proton is extremely bound (small degrees of freedom).

In terms of bound water, the content of bound water in the P2-2 group was significantly lower than that in the control group. Subsequently, the pressure of UHP gradually increased, and the content of bound water increased significantly. And the peak position of P2-2 was significantly shifted to the right, indicating that the pressure treatment of P2-2 resulted in a significant decrease in the ability of binding water and an increase in the degree of freedom of melon juice bound water. In terms of semi-bound water, the semi-bound water content of P2-2 was not significantly different from that of the control. The content of semi-bound water increased with increasing pressure. Unlike bound water, the peak position of P2-2 was significantly shifted to the left, suggesting that P2-2 made the semi-bound water more tightly bound to the macro-molecules, which decreased the free degree of semi-bound water (31). The free water trends were significantly different, with P2-2 causing a significant increase in the free water content of melon juice and a significant decrease with increasing pressure. This may be due to the fact that the pressure broke up the juice cells so that the water inside the cells was dispersed in the matrix, while the high pressure re-secured the water making it less likely to diffuse. The peak position of free water showed an overall leftward shift in the UHP treated group. The UHP treatment resulted in a slight downward trend of free water in melon juice.

The table in Figure 3 shows the area ratio of free water to bound water in melon juice, and the free water percentage of P2-2 was more than 7,000 times higher than that of the other treatment groups. There was a big gap in the current research on the effect of high pressure on the water status of fruit juices. We hypothesized that it might be due to the appropriate pressure that caused the breakage of macro-molecular cells in the juice thus releasing much bound water leading to a significant increase in the percentage of free water. The effect of UHP on the distribution state of water has also been shown in strawberry studies (12). On the other hand, too high pressure produced an asymptotic effect on the destruction of juice cells, and the higher the pressure, the degree of breaking was instead significantly reduced (6, 26, 32). This was consistent with the results for the particle size (Figure 2). P2-2 treatment can significantly enhance the percentage of free water in melon juice. Thus, it enhanced the volatilization effect of melon juice by increasing the free water and promoting the mobility of water.

### 3.4 Effect of ultrahigh-pressure treatment on the rheological properties of melon juice

**Figure 4:**
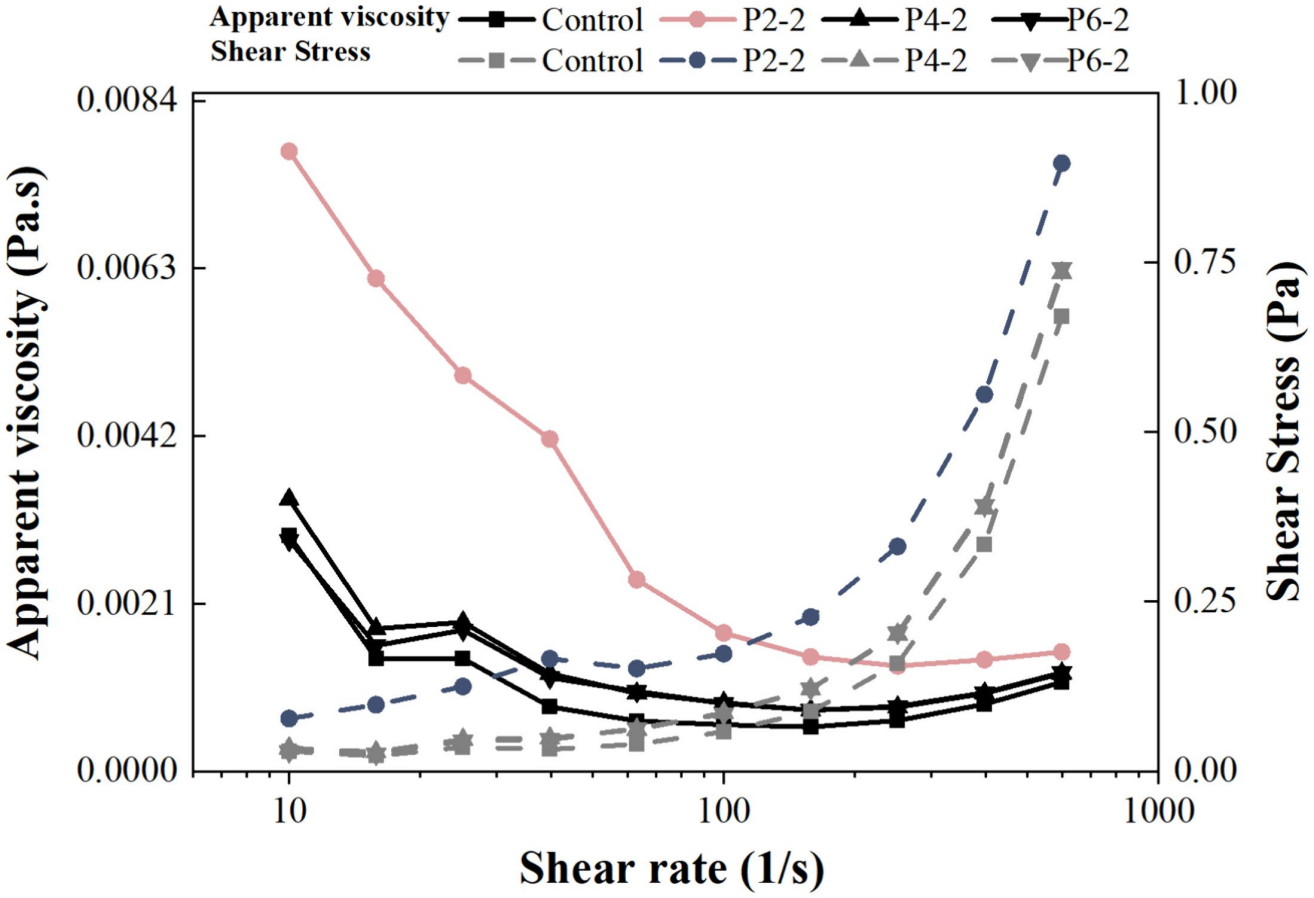
Influence of UHP pressure level on apparent dynamic viscosity and shear stress of melon juice.

The dynamic viscosity of the samples increased significantly after UHP treatment compared to fresh juice (Figure 4). The apparent dynamic viscosity of the samples varied most at the lowest shear rate and became flattened at higher shear rates. It can be concluded that fresh and UHP treated juices exhibited shear thinning behavior, which commonly occurred in fruit products (33). For example, UHP processing reduced the viscosity of tomato juice (32) and apple juice (34). It has been shown that the rheology of fruit juices was influenced by many factors such as temperature, pH, sugar type and content, particle size and composition of other dispersed particles (35). The increased apparent dynamic viscosity of juice due to UHP was hypothesized to be attributed to three factors. One may be due to the increased permeability of the cell wall induced by UHP leading to an increase in the solubility of macro-molecular carbohydrates (e.g., starch and pectin) (36). The second may be due to the possibility of UHP induced protein tissue coagulation or compaction effects (37). Thirdly, it may be due to the fact that UHP reduced the particle size of the juice, thus increased the apparent viscosity of the juice (24). This was most likely responsible for the highest apparent viscosity in the P2-2 group. This was consistent with the results of the previous particle size data (Figure 2).

Figure 4 also showed the relationship between shear stress and shear rate. The shear stress varied according to the pressure level. Between treatment groups, the trend of shear stress was consistent with the results of apparent viscosity. P2-2 was the highest, P4-2 overlapped with P6-2, and the control group was the lowest. It was hypothesized that UHP broke up the large particles in the juice, thereby increased the surface area of the juice particles and improving inter-particle interactions, thereby increasing the bond strength between the juice particles (27). Alternatively, the change in shear stress may be due to the type of processing. Similar results were found in the increase of shear stress in mango pulp after UHP treatment, but the shear stress decreased when the pulp was heat-treated (38).

### 3.5 Effect of ultrahigh-pressure treatment on differential scanning calorimetry of melon juice

**Figure 5:**
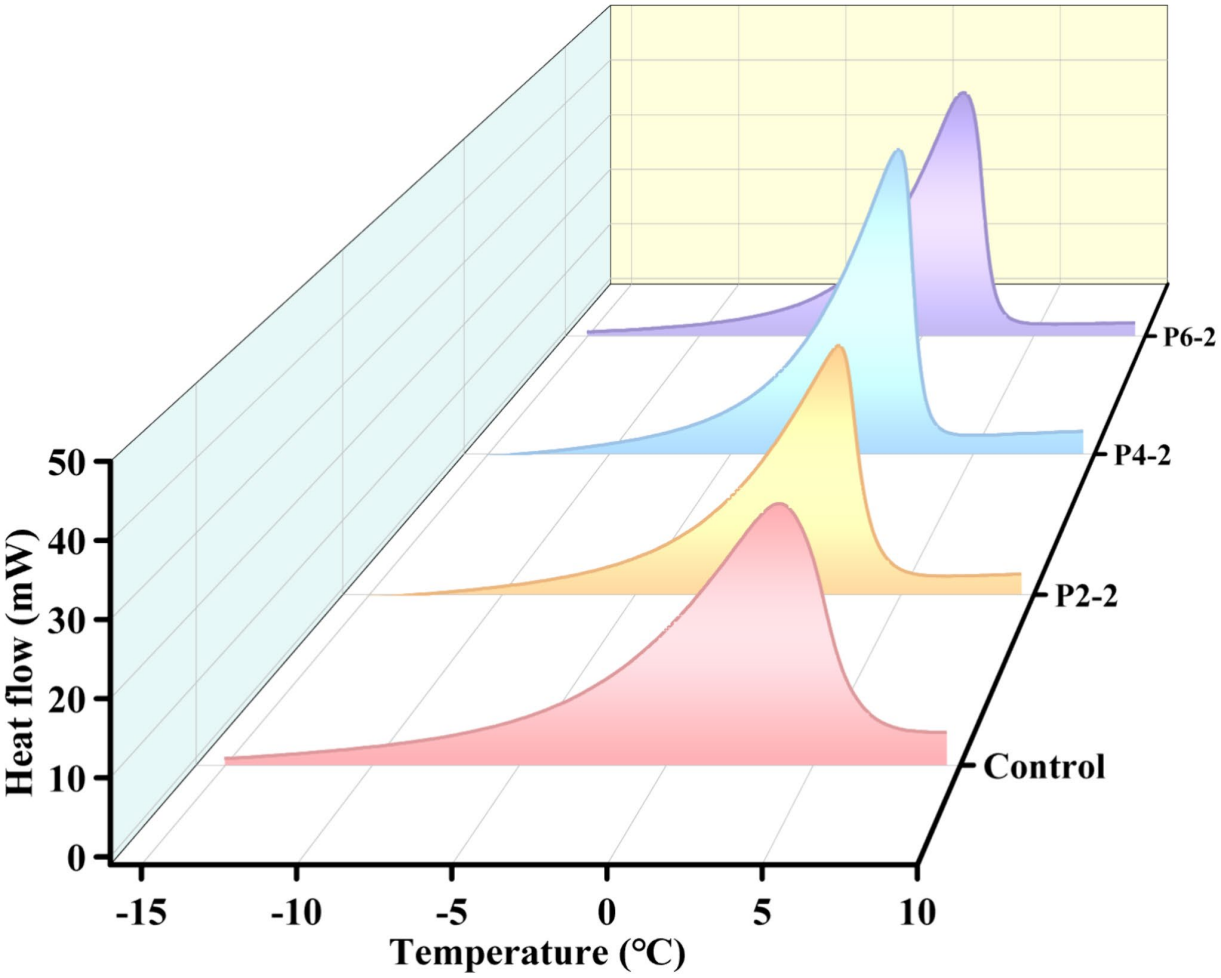
Influence of UHP pressure level on melting process of melon juice.

The effect of different pressures on the melting peak parameters of melon juice were given in Table 2, including melting onset temperature (Onset), melting end temperature (End), melting heat flow peak (Peak Height) and melting enthalpy (*ΔH*). It can be seen that as the pressure increased, the onset temperature of melting of melon juice gradually decreased and the melting temperature interval gradually shortened. The peak heat flow and the enthalpy of the melting process gradually increased. The positive value of enthalpy was related to the heat absorption behavior of the melting process. This was better shown in the melting process in Figure 5. In addition, the heat required for melting was a minimum of 198.91 J/g for the P2-2 treatment group. UHP treatment caused a decrease in the enthalpy of melting, which represented poor thermal stability (39, 40), which meant that the molecular structure of P2-2 treated melon juice was poorly stabilized, which resulted in the characteristic aromatic component of P2-2 being more volatile. This was consistent with the results of the gas–liquid partition ratios of the characteristic aromatic components. In addition, the enthalpy of melting (*ΔH*) gradually increased with increasing UHP pressure. This was consistent with the thermal behavior of black garlic melanoidins treated with high pressure. The reason for this was hypothesized to be related to the disruption of structural and non-covalent bonding interactions of the polymer and the reorganization of intramolecular and intermolecular bonds induced by the UHP treatment (41). UHP modified chemical bonding through strong shear forces, leading to changes in macromolecular structure. Significant structural changed in juice polymers may affect both hydrogen bonding and related ionic and hydrophobic interactions. However, there was limited information on the chemistry behind these effects.

**Table 2:** Melting peak heat flow parameters of melon juice under different pressure level.

Treatments	Onset (°C)	End (°C)	Peak height (mW)	*ΔH* (J/g)
Control	−2.34 ± 0.21^a^	6.37 ± 0.45^a^	30.68 ± 2.13^d^	212.89 ± 4.63^c^
P2-2	−2.60 ± 0.34^ab^	3.95 ± 0.32^b^	34.39 ± 1.98^c^	198.91 ± 3.58^d^
P4-2	−2.86 ± 0.47^b^	3.33 ± 0.53^b^	40.66 ± 2.56^b^	235.24 ± 2.95^b^
P6-2	−2.87 ± 0.14^b^	2.92 ± 0.02^c^	46.70 ± 2.64^a^	240.95 ± 3.25^a^

Results are presented as mean ± standard deviation (*n* = 3). Different lowercase letters following the values within the same column indicate significant differences (*p* < 0.05).

### 3.6 Effect of ultrahigh-pressure treatment on isothermal titration calorimetry of melon juice

**Figure 6:**
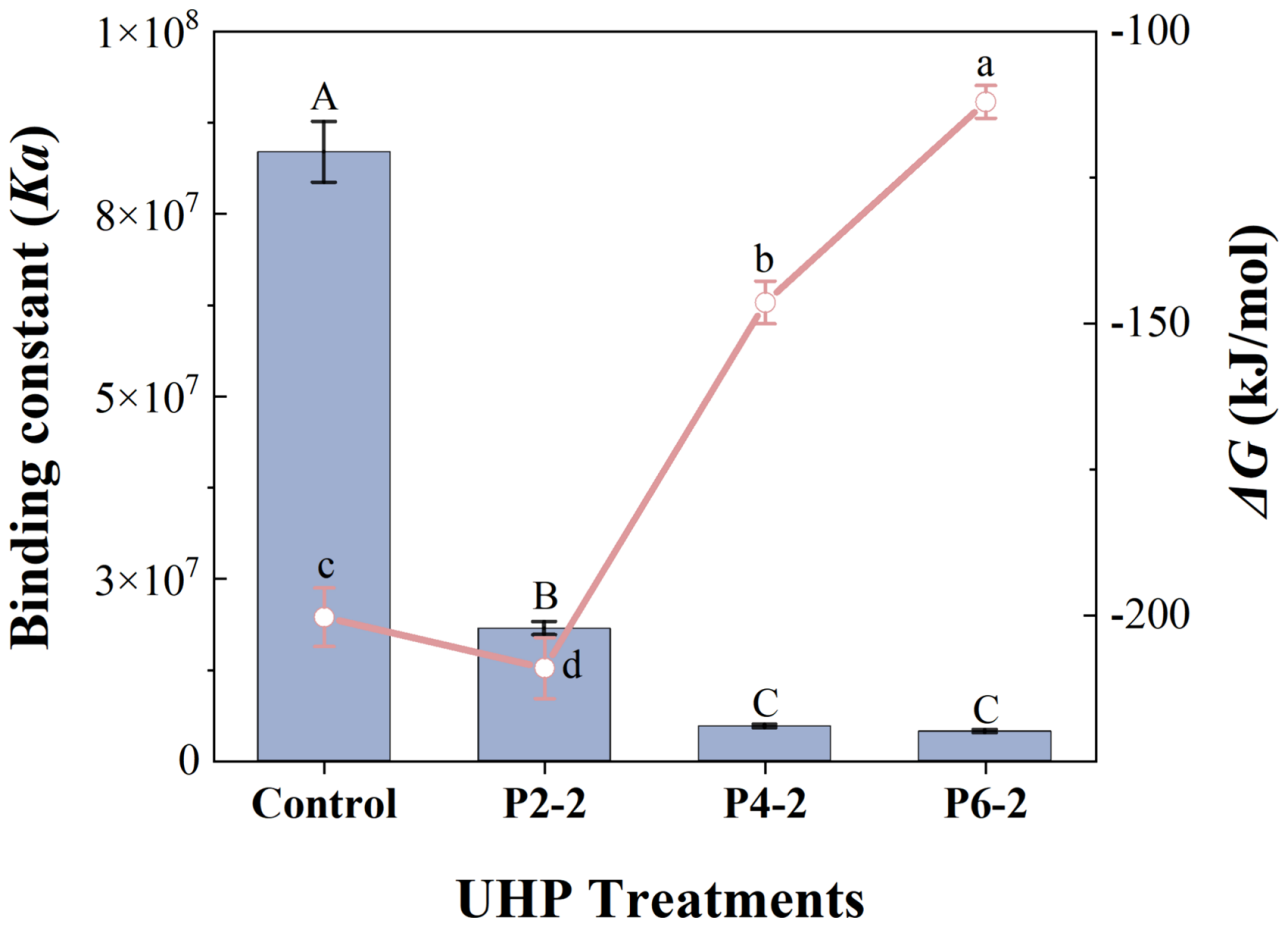
Influence of UHP pressure level on binding constant (*Ka*) and gibbs free energy (*ΔG*) of melon juice. Histogram, binding constant (*Ka*); line graph, gibbs free energy (*ΔG*). Histogram with capital letters represent significant differences and line graph with lowercase letters represent significant differences (*p* < 0.05).

Figure 6 showed the effect of UHP pressure on the binding constant (*Ka*) and Gibbs free energy (*ΔG*) of melon juice. The Ka of melon juice was significantly reduced after UHP treatment, which means that high pressure leads to weaker intermolecular binding of melon juice. And with the increase of pressure, the effect of UHP on *Ka* also appeared a gradual effect. That is, as the pressure increased, the degree of effect on *Ka* gradually decreased. The most significant reduction in *Ka* was observed for P2-2. Combined with the previous conclusions such as particle size, it indicated that UHP had the greatest effect on the intermolecular force of melon juice at a treatment pressure of 200 MPa.

Isothermal titration calorimetry (ITC) was based on the measurement of the heat generated during intermolecular binding, which means that the high pressure treatment intensifies the intermolecular interactions in melon juice. *ΔG* referred to the portion of the system’s reduced internal energy that can be converted into external work during a given thermodynamic process. *ΔG* is related to *ΔH*, the thermodynamic temperature (*T*) and the entropy change (*ΔS*), i.e., *ΔG = ΔH - TΔS*. Enthalpy changed due to hydrophobic effects, hydrogen bonding, π-π and van der Waals interactions, while entropy changed due to conformational changes and desolvation (42). *G* < 0 meant that the reaction could proceed autonomously, mainly from positive entropy contributions and smaller enthalpy contributions. It was reasonable to speculate that the intermolecular interactions in melon juice were mainly driven by entropy changes due to conformational changes and desolvation. This conformational change process may involve the reorganization of sugars and volatile compounds in melon juice (19). Moreover, the overwhelming driving force in the sugar-volatile binding was the hydrophobic effect. Although about half of the hydrophobic effect originated from the entropy of the hydrogen-bonded three-dimensional network lattices of water molecules at room temperature, hydrophobic effect still had an enthalpic component (43). This may indicated a potential contribution of hydrogen bonding and hydrophobic effects in the influence of UHP on the intermolecular interactions of melon juice, but this still needs further validation.

The *ΔG* represented the magnitude of the intermolecular binding strength (20). The smallest *ΔG* of P2-2 represented the most unstable structure of P2-2 and the strongest degree of volatilization. This was consistent with the results of the gas–liquid partition ratio of the characteristic aromatic components of melon juice (Figure 1). It was also suggested that UHP led to enhance volatility of the P2-2 characteristic aromatic component by altering the intermolecular *Ka* and *ΔG* of melon juice thereby causing conformational change and desolventization of the juice.

## 4 Conclusion

The advantageous impact of UHP on preserving the flavor of fruit and vegetable juices has been acknowledged by the majority of researchers. However, there is still no definitive conclusion regarding the mechanism of UHP in maintaining the flavor of fruit and vegetable juices. In this current investigation, a new approach was undertaken from a physics perspective. The primary finding revealed that among the UHP treatment groups, the P2-2 group exhibited the smallest and most uniform melon juice particle size, with a free water content more than 7,000 times higher than that of the other groups. This significantly reduced the *ΔH*, *Ka*, and *ΔG* of the melon juice, resulting in greater volatility of the P2-2 group, which significantly enhanced the characteristic aromatic concentration of the melon juice in the gas phase.

In our previous research, we hypothesized that UHP treatment increased the concentration of characteristic aromatic components in melon juice in the gas phase, possibly due to the reduction of liquid surface tension. In alignment with the main findings of this study, the P2-2 treatment group under UHP showed a significant reduction in the particle size of melon juice. There was an observed relationship between the liquid surface tension and particle size, typically a positive correlation – where excessively small particles may not effectively interact with the liquid molecules, leading to a reduction in liquid surface tension. Therefore, the mechanism by which UHP increased the aromatic components of melon juice may be attributed to the reduction in particle size and subsequent decrease in surface tension, leading to increased volatilization of the aromatic components. Specific characterization we further demonstrated by the determination of free water content, *ΔH*, *ΔG*, and *Ka* in the P2-2 treatment group of melon juice. However, with increasing pressure, asymptotic behaviors were observed for particle size, free water content, and *ΔG*. The results showed that the effect of pressure on the volatile energy of melon juice was very complex, and the important factors involved need to be explored at a deeper level.

The volatilization properties of pure inhomogeneous systems were closely related to the nature of their gas–liquid interface, the type of molecules and their molecular structure. These properties were generally affected by the strength of cohesion between molecules, which was determined by the nature of the intermolecular bonds. Therefore, the investigation of the mechanism of the effect of UHP on liquid flavor may start from the intermolecular bond energy and force in the future. The study of a series of reorganizations of juice intramolecular and intermolecular bonds affected by UHP through strong shear forces will be an interesting entry point to elucidate the mechanism of UHP flavor enhancement in juices.

## Data availability statement

The original contributions presented in the study are included in the article/supplementary material, further inquiries can be directed to the corresponding author.

## Author contributions

XL: Methodology, Writing – original draft, Writing – review & editing. FL: Methodology, Writing – review & editing. BW: Investigation, Validation, Writing – review & editing. FR: Writing – review & editing. WW: Funding acquisition, Resources, Writing – review & editing. CZ: Funding acquisition, Resources, Writing – review & editing.
